# Genome sequences of *Microvirga* spp. CF3062 and CF3016 isolated from nodules found on herbarium specimens collected in 2004 and 2015

**DOI:** 10.1128/mra.00161-24

**Published:** 2024-11-05

**Authors:** Renee H. Petipas, Hanna Kehlet-Delgado, Amanda A. Antoch, Maren L. Friesen

**Affiliations:** 1 Department of Plant Pathology, Washington State University, Pullman, Washington, USA; 2 Department of Microbiology, University of Washington, Seattle, Washington, USA; University of Strathclyde, Glasgow, United Kingdom

**Keywords:** *Microvirga*, historical strains, genome, nodule-associated bacteria

## Abstract

We present the genomes of two *Microvirga* isolates isolated from nodules found on *Medicago lupulina* herbarium specimens at the Marion Ownbey Herbarium at Washington State University. These genomes and others from herbarium specimens offer an unprecedented opportunity to study the bacterial evolution of plant-associated microbes over long time scales.

## ANNOUNCEMENT

*Microvirga* spp. CF3062 was isolated from a nodule found on a herbarium specimen of the legume *Medicago lupulina*, collected in Klickitat County, Washington in 2004. *Microvirga* spp. CF3016 was isolated from a specimen of *Medicago lupulina* in Grant County, Washington in 2015. Plant specimens are at the Marion Ownbey Herbarium (specimen ID 390849 and 390614, respectively). *Microvirga* are known constituents of the nodule microbiome (1) and even nodulate and fix nitrogen (2–6). Here, we present the draft genomes of two *Microvirga*, CF3062 and CF3016.

**Fig 1:**
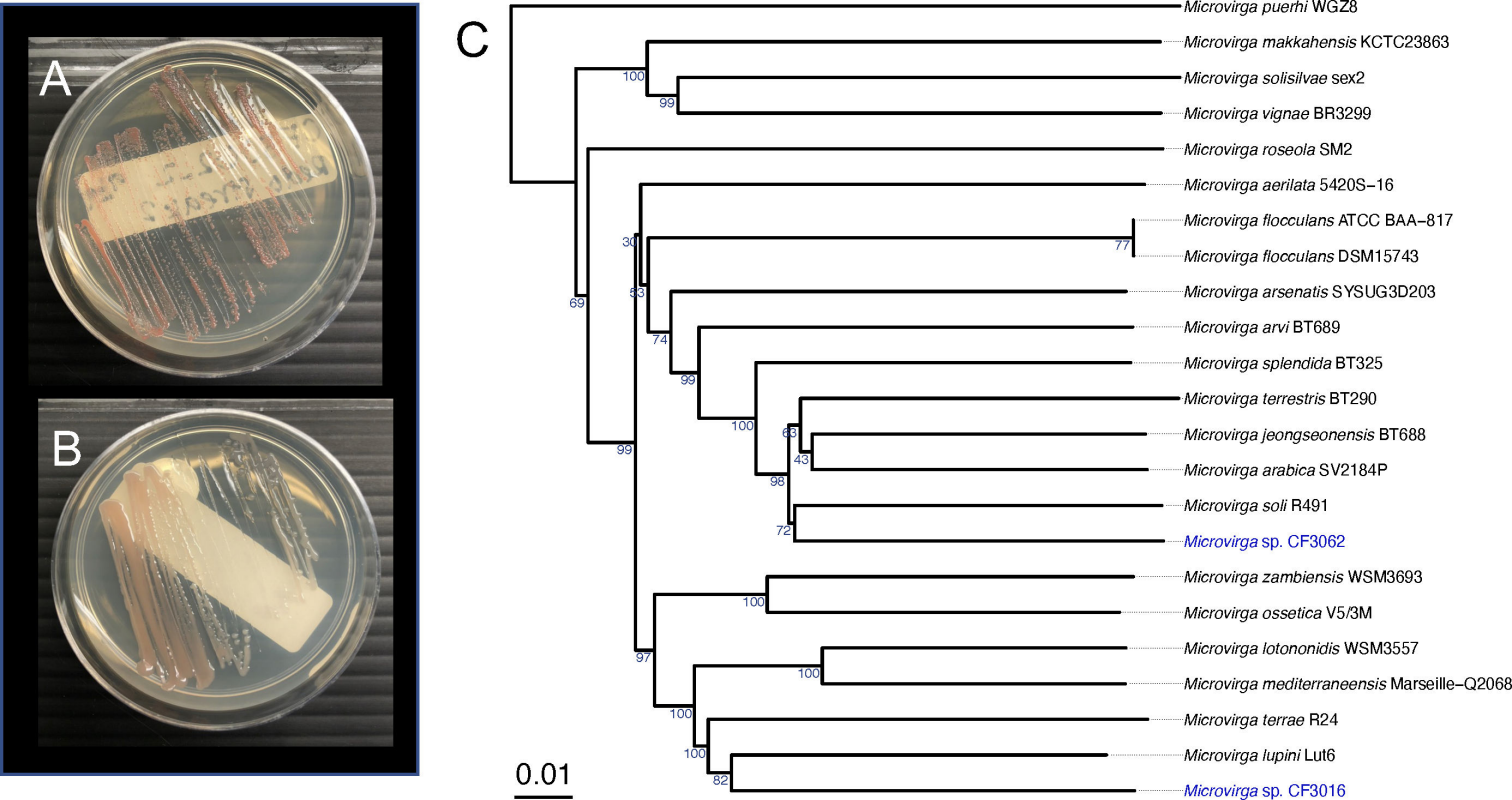
Tryptone yeast cultures of *Micovirga* spp. CF3062 (**A**) and CF3016 (**B**) isolated from nodules found on *Medicago lupulina* herbarium specimens at the Marion Ownbey Herbarium at Washington State University. Phylogeny (**C**) inferred with FastMe version 2.1.6.1 (8) from GBDP (Genome BLAST Distance Phylogeny) genome distances (9, 10) within the Type Strain Genome Server version 391 (9, 11) of *Microvirga* species showing the placement of CF3062 and CF3016. The node value numbers are GBDP pseudo-bootstrap support values from 100 replications. Tree was midpoint rooted (12) and viewed with ggtree version 3.10.0 (13).

Historic nodules were rehydrated in filter-sterilized 10% sucrose solution for 48 hours, rinsed in autoclaved, filtered water (Milli-Q) seven times, and crushed in 200 µL 1/2× phosphate-buffered saline before plating. CF3062 was originally isolated onto yeast mannitol (YM) plus calcium, and CF3016 was originally isolated onto YM plus sodium chloride (NaCl) and incubated at 30°C for 12 hours before being observed for growth. Media modifications are detailed in reference (7). We transferred bacteria at least twice to ensure isolation. After recovery from frozen stocks (−80°C), plating on tryptone yeast, and incubation at 30°C for 24 hours, a single colony was selected to be grown under the same conditions for DNA (gDNA) extraction (Fig. 1A and B). DNA was extracted using the DNeasy PowerLyzer Microbial Kit (Qiagen, Germantown, MD, USA). DNA template was purified using Sera-Mag Speedbeads (Thermo Fisher Scientific, Waltham, MA, USA) and eluted in tris buffer. Library preparation and 250 Mbp Illumina whole-genome sequencing were done at SeqCenter (Pittsburgh, PA, USA).

DNA libraries were prepared using the Illumina DNA Prep Kit and IDT 10 bp UDI indices and sequenced on an Illumina NextSeq 2000, producing 2 × 151-bp reads. Demultiplexing, quality control, and adapter trimming were performed with bcl2fastq (version 2.17; Illumina). Reads were assembled with Spades (version 3.14.1) (14) within Shovill (version 1.1.0) (15), with the following options: genome size, 7.5 Mb; minimum contig length, 500 bp; and minimum coverage, 20. Final assemblies were annotated with PGAP version 6.6 (16). QUAST version 5.2.0 (17) and CheckM version 1.0.12 (18) were used to assess genome quality and completeness. We used the Type Strain Genome Server version 391 (9, 11) to compare CF3062 and CF3016 to the genomes of related type strains by obtaining a Genome BLAST Distance Phylogeny and genome-relatedness indices including digital DNA-DNA hybridization analysis (dDDH) (9, 10). We used antiSMASH version 7.1.0 (19) for mining genes with potential biosynthetic functions. Average nucleotide identity (ANI) was calculated with FastANI version 1.33 (20). Default parameters were used unless otherwise noted.

Genome statistics can be seen in Table 1. ANI between CF3016 and CF3062 is 84.64%. The maximum dDDH values, estimated by Genome-to-Genome Distance Calculator formula d_4_ (10), are 33.0% with type strain *Microvirga lupini* Lut6 (accession GCA_000517005) and 34.6% with type strain *Microvirga jeongseonensis* BT688 (accession GCA_014699075) for CF3016 and CF3062, respectively. These values are below the recommended dDDH threshold for species-level delineation of 70% (21, 22), indicating CF3016 and CF3062 each belong to potentially novel *Microvirga* species (Fig. 1C). antiSMASH revealed the biosynthetic potential for homoserine lactones, terpenes, and Type III polyketide synthase in both CF3062 and CF3016. Genes involved in biological nitrogen fixation (*nodABC* and *nifH*) were not found in either genome.

**TABLE 1:** Genome statistics for two *Microvirga* isolates from herbarium nodules

Strain ID	CF3016	CF3062
Genome size (bp)	4,769,693	4,172,190
Raw reads	5,036,218	4,737,016
Number of coding sequences (with protein)	4,430	3,875
Number of RNAs	59	56
Number of contigs	30	28
GC content (%)	63.51	62.63
Contig *L*_50_	5	3
Contig *N*_50_ (bp)	360,743	403,870
Sequence read archive accession	SRR27329153	SRR27329152
GenBank accession	JAYGGM000000000	JAYGGN000000000
Completeness	99.06%	98.75%
Contamination	0.10%	0.31%

## Data Availability

Whole-genome data can be downloaded under genome accession numbers JAYGGM000000000 and JAYGGN000000000 and sequencing read archive numbers SRR27329153 and SRR27329152 for CF3016 and CF3062, respectively. The project data can be found under BioProject accession no. PRJNA1055671.
